# Genome-wide association study of idiopathic epilepsy in the Italian Spinone dog breed

**DOI:** 10.1371/journal.pone.0315546

**Published:** 2025-03-05

**Authors:** Christopher A. Jenkins, Luisa De Risio, Artitaya Lophatananon, Thomas W. Lewis, Donna Foster, Jim Johnson, Hannes Lohi, Cathryn S. Mellersh, Sally L. Ricketts

**Affiliations:** 1 Department of Veterinary Medicine, Canine Genetics Centre, University of Cambridge, Cambridge, United Kingdom (Formerly at the Animal Health Trust, Newmarket, Suffolk, United Kingdom),; 2 Division of Population Health, Health Services Research & Primary Care, University of Manchester, Manchester, United Kingdom; 3 Neurology/Neurosurgery Service, Centre for Small Animal Studies, Animal Health Trust, Newmarket, Suffolk, United Kingdom; 4 Linnaeus Veterinary Ltd, Shirley, Solihull, United Kingdom; 5 The Kennel Club, London, United Kingdom; 6 School of Veterinary Medicine and Science, University of Nottingham, Sutton Bonington, United Kingdom; 7 Department of Medical and Clinical Genetics, University of Helsinki, Helsinki, Finland; 8 Department of Veterinary Biosciences, University of Helsinki, Helsinki, Finland; 9 Folkhälsan Research Center, Helsinki, Finland; West Bengal University of Animal and Fishery Sciences, INDIA

## Abstract

Idiopathic epilepsy (IE) has a high prevalence and a severe clinical course in the Italian Spinone breed of dog. A genome-wide association study meta-analysis of 52 cases and 51 controls was conducted to identify genomic regions that may be involved with the development of IE. Subsequent to the meta-analysis, a set of 175 controls and an independent validation set of 23 cases and 23 controls were genotyped for SNPs showing suggestive association with IE to find variants exhibiting evidence of replicable association and to test the predictiveness of SNPs for IE status when combined in a weighted risk score. Although two regions showed statistically significant association with IE in the GWAS meta-analysis, and additional regions with suggestive association were identified, the findings were not emulated in the validation set. This is the first GWAS of IE in the Italian Spinone, and the findings suggest that IE in the breed is not monogenic and demonstrates the challenges when investigating a multigenic or complex inherited disease in a numerically small domesticated animal population.

## Introduction

Epilepsy is common in dogs, with an estimated prevalence of 0.62%–0.75% in the general dog population [1,2] and a much higher prevalence reported for some breeds [3–7]. IE in the Italian Spinone breed of dog has been clinically characterised and defined as two or more epileptic seizures (occurring > 24 hour apart) with an onset between 6 months and 6 years of age in dogs with normal interictal physical and neurologic examinations and unremarkable results of haematology and serum biochemistry [6]. This breed-wide survey of 1,192 UK dogs born between the years 2000 and 2011 estimated the prevalence of IE in the Italian Spinone breed to be 5.3% (95% confidence interval 4.0–6.6%). The age of onset in the 63 cases identified in the study ranged from 11 months to six years (median 2.9 years) and their seizures were found to be typically generalised tonic-clonic, with approximately half of dogs reported to have seizures with a focal onset that became generalised. IE has been reported to be particularly severe in this breed, with cluster seizures reported for the majority of cases; status epilepticus occurring in over a fifth of cases; and a 32% mortality due to epilepsy-related causes [6].

The consensus based on current evidence is that for common human diseases with an adult-onset, heritability is conferred by many genetic variants acting together that range from low-frequency with high-effect on disease risk, to common with low-effect on risk [8]. Each single genetic variant does not give a strong indication of an individual’s overall disease risk, but a polygenic risk score (PRS) incorporating multiple variants can be used. These are typically calculated as the total number of risk alleles that an individual carries, weighted by the measured effect of each of the variants [8]. Following GWAS and replication studies for several complex human diseases, PRS have been developed to help identify individuals at highest risk of disease in the population and who therefore may benefit from clinical and/or lifestyle interventions [8,9]. PRS have been produced for multiple human diseases, including coronary heart disease [8–12] and some types of cancer [13–15].

The present study sought to identify genetic variants that are reproducibly associated with IE in the Italian Spinone dog breed and to assess their utility as a predictive tool in a genetic risk score in an independent set of Spinoni. We performed a GWAS meta-analysis, utilising genotype imputation, followed by replication testing in additional samples.

## Materials and methods

### Sample collection

Idiopathic epilepsy cases were recruited through a breed-wide survey as reported previously [6]; owner reported questionnaires; or were cases diagnosed by veterinary neurologists in the neurology unit at the Animal Health Trust Centre for Small Animal Studies, Newmarket, UK (led by LDR) or Linnaeus referral veterinary hospitals. All Italian Spinoni affected by IE recruited as outlined above were diagnosed by LDR (a board-certified veterinary neurologist) through review of the owner questionnaire, medical records, and epileptic seizure video footage when available. The diagnosis of IE was based on the criteria set by the IVETF [16]. In addition to the above, four dogs (two cases and two controls) included in the study were collected by the University of Helsinki, Finland (cases were diagnosed by a veterinary neurologist). All controls were dogs over the age of seven years that were reported by their owners to have never had a seizure.

Two GWAS datasets were generated for this study, genotyped four years apart; Set 1 (29 cases, 29 controls) and Set 2 (23 cases, 22 controls). The controls in Set 2 were selected at random from a large pool (approximately 170) of available control samples, and the relatedness of the cases and controls from the UK were compared to the Kennel Club-registered Italian Spinone population (as outlined below) to ensure that the cohorts did not have an over-representation of closely related individuals. Due to DNA availability, a subset (12 cases, 16 controls) of the 32 UK dogs investigated for relatedness were combined with predominantly non-UK dogs to make up the final Set 2. A set of 175 control dogs over the age of seven years were used as a ‘semi-replication’ set to investigate the most significantly associated SNPs from the GWAS. A subsequently developed independent validation set consisted of 23 cases and 23 controls. Additional details of the dogs included in the four sets are given in **S1 Table** and **S1 Fig**.

Samples were collected in the form of buccal swabs or whole blood residual from routine clinical tests (Animal Health Trust Clinical Research Ethics Committee Project No. 73-2016, University of Cambridge Department of Veterinary Medicine Ethics and Welfare Committee No. CR512). Sample collection in Finland was ethically approved by the Animal Ethics Committee of State Provincial Office of Southern Finland (ESAVI/25696/2020). DNA was extracted from swabs using a QIAamp DNA Blood Midi Kit (Qiagen), and blood samples using a standard chloroform protocol or a semi-automated Chemagen extraction robot (PerkinElmer Chemagen Technologie, Waltham, MA, USA).

### Kinship calculations and selection of controls for Set 2

At the time of genotyping for Set 2, DNA samples of approximately 170 controls were available. A set of controls was selected at random for GWAS from the available dogs; however, we sought to confirm that bias had been avoided in the selection, and that the subset of the potential Set 2 controls (that were UK-Kennel Club registered) were representative of a wider population of Italian Spinoni in a contemporaneous UK Kennel Club-registered population. The relatedness of the potential Set 2 (21 cases and 25 controls that were UK-Kennel Club registered) was compared to 1,000 randomly selected samples of the same number of dogs in each group from the Kennel Club pedigree database during 2017. See **S1 File** and **S2 Table** for a more detailed description of the methodology used and the results of this analysis. The mean kinships among and between the case and control cohorts were found to be representative of random samples of dogs from similar birth years (**S1 File** and **S2 Table**).

### Array genotyping

The two study sets were genotyped on different arrays: Set 1 on the Illumina CanineHD BeadChip (173,662 SNPs) and Set 2 using the Axiom Canine HD array (up to 729,642 SNPs and other DNA variants). High-quality genotyping data were available for 29 cases and 29 controls (individual call rate > 95%) for Set 1. For Set 2, after quality control using the Axiom Analysis Suite and the Best Practices Workflow, genotype data were available for 23 cases and 22 controls with individual call rate > 90%.

A subset of the validation set, consisting of 18 cases and 18 controls, was subsequently genotyped on the Illumina CanineHD BeadChip and high-quality genotype data were obtained for all dogs.

### Genotype imputation and GWAS meta-analysis

Genome-wide genotype imputation was carried out for Set 1, as described previously [17], to allow meta-analysis with Set 2 whilst maximising the number of SNPs available for analysis. Imputation increased the SNP density of Set 1 to Axiom-array level; to 476,313 autosomal SNPs (imputation accuracy: mean R^2^ = 0.97, mean genotype concordance 98.2%) [17]. The validation set was imputed to Axiom-array level using the same reference panel used for Set 1. The imputed data were converted to BIMBAM format for downstream analysis.

GWAS analysis was conducted for each dataset independently using GEMMA, with a linear mixed model to correct for population stratification [18]. SNPs were filtered to remove those with a Hardy-Weinberg P-value < 5 x 10^−5^; those with a SNP call rate < 97%; or with a minor allele frequency (MAF) < 5%. Set 1 SNPs were also filtered to exclude those with an IMPUTE2 ‘Info’ statistic (imputation certainty) < 0.5 [19,20]. After quality control 341,811 and 358,527 SNPs remained for Set 1 and Set 2, respectively.

Fixed-effects meta-analyses were carried out using the statistical software package Stata (Stata 15. College Station, TX, USA), for the SNPs common across the GWAS sets, using the summary statistics produced by GEMMA. Heterogeneity was assessed using the Q statistic.

A two-dimensional multidimensional scaling (MDS) plot was generated for Set 1, Set 2, and the validation set using PLINK (**S2 Fig**). Prior to MDS analysis, the datasets were filtered by SNP call rate (< 97%), individual call rate (< 90%), and Hardy-Weinberg equilibrium P-value (< 5 x 10^−5^). Set 2 and the validation set were also filtered by the IMPUTE2 ‘info’ statistic (< 0.5). To allow a combined MDS analysis the three genotype datasets were merged using PLINK, keeping only the SNPs present in all.

### Genotyping selected GWAS SNPs in additional dogs

Genotyping of individual SNPs in the semi-replication set of 175 control dogs, and the validation set, was carried out using allelic discrimination assays, utilising an ABI StepOne real-time thermal cycler and LUNA Universal Probe qPCR Master Mix (NEB). The sequences of the TaqMan SNP genotyping assay (ThermoFisher) primers and probes (reporters) are in **S3 Table**. A fast ramping speed was used, and the following cycling conditions: 30 seconds pre-PCR read at 25°C, 3 minute 95°C holding stage, 40 cycles of 95°C for 3 seconds and 10 seconds at 60°C, followed by a 25°C post-PCR read stage for 30 seconds.

### Chi-square analysis of SNPs in additional control dogs

Genotypes of the most significantly associated SNPs in the 10 regions identified in the meta-analysis were extracted from the array genotype and imputed genotype data for dogs included in the GWAS meta-analysis. Imputed genotype probabilities were converted to hard genotype calls using PLINK (v1.90) [21], and genotype calls with uncertainty > 0.1 were treated as missing. Genotype calls for the 10 SNPs were obtained through allelic discrimination assays, as outlined above, for the semi-replication set of 175 control dogs. Chi-square analysis was caried out using Stata, with and without the inclusion of the 175 additional controls.

### Testing a five-SNP genetic risk score in an independent study set

Weighted five-SNP risk scores were calculated as follows:


$$5\times \frac{\beta 1\times SNP1+\beta 2\times SNP2+\beta 3\times SNP3+\beta 4\times SNP4+\beta 5\times SNP5}{\beta 1+\beta 2+\beta 3+\beta 4+\beta 5}$$

Where ‘SNP’ represents the count of the risk-conferring allele, and ‘β’ the beta-coefficient from the meta-analysis, for each of the five SNPs. If the meta-analysis-derived beta-coefficient was calculated for the non-risk allele, and therefore negative, the allele was ‘flipped’ to produce a positive beta-coefficient coded for the risk allele (× −1).

Genetic risk scores were calculated for dogs included in the GWAS meta-analysis using the array genotype data or imputed genotype data. Imputed genotype probabilities were converted to hard genotype calls as above. Genotype calls from allelic discrimination assays were used to generate genetic risk scores for dogs that were part of the validation set. Individuals were excluded from the analysis if genotype calls were missing for any of the five SNPs.

To assess genetic risk score performance in each set of dogs the receiver operating characteristic (ROC) and area under the curve (AUC) were calculated and plotted using logistic regression, for IE case/control status and the genetic risk score, and using Stata’s ‘lroc’ command. Calibration plots were generated using the ‘pmcalplot’ Stata module to further evaluate the predictive performance of the genetic risk score.

## Results

### GWAS meta-analysis identifies loci showing suggestive association with IE

**Fig 1:**
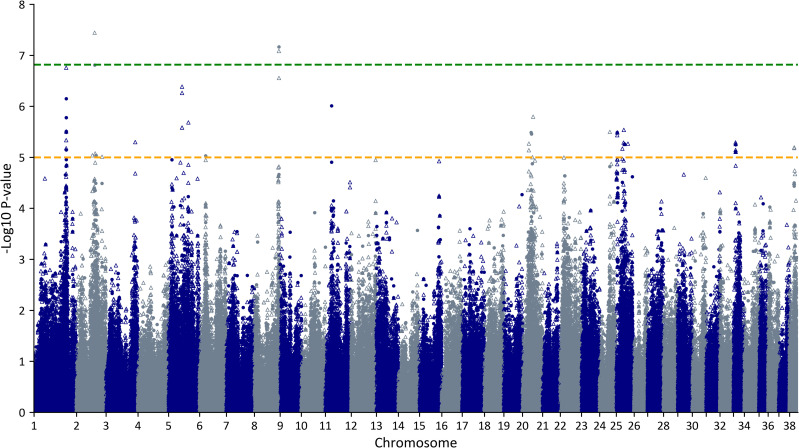
GWAS meta-analysis of 52 Italian Spinone idiopathic epilepsy cases and 51 controls (328,622 SNPs). Plot of negative log (base 10) transformed P-values. X-axis is SNP location by chromosome (left to right, autosomes 1 to 38). Solid circles indicate array-genotyped SNPs, hollow triangles denote SNPs imputed for Set 1. Green (upper) line shows the Bonferroni-corrected threshold for statistical significance (P < 1.5 x 10^−7^). Orange (lower) line indicates the threshold for suggestive association (P <  1 x 10^−5^).

A meta-analysis of two GWAS datasets, Set 1 and Set 2, including a total of 52 cases and 51 controls and comprising data from 328,622 SNPs, identified SNPs in two regions that passed the Bonferroni corrected threshold for statistical significance (1.5 x 10^−7^); on chromosomes 2 and 8 (**Fig 1 and Table 1**). Suggestive associations also indicated that multiple other regions might have a role in conferring disease risk in this breed. SNPs with P-values <1 x 10^−5^ were identified on 10 additional chromosomes (**Fig 1 and Table 1**). Two of the 12 SNPs, on chromosomes 33 and 38, were excluded from further analysis due to high heterogeneity (P-value ≤ 0.01) between the two study sets (**Table 1** and **S3 Fig**).

**Table 1:** The most significantly associated GWAS meta-analysis SNPs on each chromosome with P-values < 1 x 10^−5^.

Genomic position ^a^	Nearest known gene	ca/co ^b^ (n)	Odds ratio (95% CI ^c^)	P-value	P-value for het. ^d^	Alleles (risk/non-risk)	Risk allele freq. (ca/co ^b^)		
**Set 1**	**Set 2**	**Validation set** ^e^
1:93123836	*JAK2*	52/51	1.40 (1.24–1.59)	1.8 x 10^−7^	0.03	T/C	0.84/0.47	0.67/0.43	0.64/0.50
2:52390106	*MAST4*	52/51	1.38 (1.23–1.54)	3.6 x 10^−8^	0.03	C/T	0.50/0.34	0.70/0.16	0.36/0.39
3:84100359	*RBPJ*	52/51	1.41 (1.22–1.63)	5.0 x 10^−6^	0.98	C/G	0.34/0.10	0.39/0.11	0.31/0.17
5:38884749	*TEKT3*	52/50	1.52 (1.29–1.79)	4.1 x 10^−7^	0.70	T/C	0.27/0.04	0.37/0.10	0.28/0.14
6:18142628	*CORO1A*	52/51	1.41 (1.21–1.64)	9.4 x 10^−6^	0.36	G/A	0.48/0.22	0.46/0.25	0.36/0.22
8:70681185	*RCOR1*	52/51	1.42 (1.25–1.62)	6.9 x 10^−8^	0.56	G/T	0.83/0.52	0.78/0.52	0.67/0.56
11:17811231	*SLC27A6*	52/51	1.46 (1.26–1.70)	9.8 x 10^−7^	0.71	T/C	0.84/0.62	0.91/0.61	0.83/0.69
20:30846012	*FHIT*	51/51	1.39 (1.22–1.59)	1.6 x 10^−6^	0.19	A/G	0.69/0.50	0.75/0.36	0.58/0.42
24:29341230	*LPIN3*	52/51	1.55 (1.29–1.87)	3.2 x 10^−6^	0.04	T/C	0.24/0.16	0.33/0.05	0.25/0.25
25:22038367	*GALNTL6*	52/51	1.81 (1.41–2.31)	2.9 x 10^−6^	0.24	T/A	0.10/0.05	0.22/0.00	0.06/0.11
33:5990467	*COL8A1*	52/51	1.37 (1.20–1.56)	5.1 x 10^−6^	8.5 x 10^−3^	A/G	0.62/0.46	0.78/0.39	Not tested
38:13596806	*TGFB2*	52/51	1.31 (1.16–1.47)	6.4 x 10^−6^	7.4 x 10^−3^	G/C	0.48/0.39	0.67/0.18	Not tested

^a^CanFam3.1 genomic location of the SNP in the format chromosome: base pair position.

^b^case / control.

^c^95% confidence intervals.

^d^heterogeneity.

^e^Risk allele frequencies in the 18 cases and 18 controls of the validation set, which was not part of the GWAS meta-analysis.

### Genotyping the strongest associated SNPs in additional controls

The 10 SNPs identified in the GWAS meta-analysis were initially genotyped in up to 175 additional unaffected Italian Spinone dogs over the age of seven years. Five of the 10 SNPs, on chromosomes 1, 2, 11, 20, and 25, showed stronger statistical associations with IE after the addition of these controls (**Table 2**).

**Table 2:** Initial replication analysis of 10 SNPs showing suggestive association with IE in the GWAS.

Genomic position ^a^	GWAS (n cases/n controls)	Genotypes ^b^ (cases/controls)			P-value (GWAS dogs)	Control set (n)	P-value (GWAS + controls)	Genotypes ^b^ (cases/controls)		
		0	1	2				0	1	2
**1:93123836**	**52/51**	3/14	18/28	31/9	**2.3 x 10** ^**−5**^	**174**	**1.7 x 10** ^**−6**^	3/58	18/112	31/55
**2:52390106**	**52/51**	11/28	21/19	20/4	**1.1 x 10** ^**−4**^	**174**	**3.4 x 10** ^**−5**^	11/109	21/84	20/32
3:84100359	52/51	20/40	26/11	6/0	8.5 x 10^−5^	175	2.0 x 10^−3^	20/138	26/81	6/7
5:38884749	50/48	22/42	26/6	2/0	3.2 x 10^−5^	175	1.1 x 10^−3^	22/155	26/57	2/11
6:18142628	52/51	10/28	35/22	7/1	3.4 x 10^−4^	175	0.02	10/91	35/112	7/23
8:70681185	52/51	1/9	18/31	33/11	3.0 x 10^−5^	173	1.7 x 10^−4^	1/33	18/115	33/76
**11:17811231**	**52/51**	0/5	13/29	39/17	**5.2 x 10** ^**−5**^	**175**	**1.2 x 10** ^**−5**^	0/19	13/118	39/89
**20:30846012**	**51/51**	3/12	23/33	25/6	**8.1 x 10** ^**−5**^	**174**	**2.3 x 10** ^**−5**^	3/59	23/120	25/46
24:29341230	52/51	24/40	27/11	1/0	2.8 x 10^−3^	175	6.9 x 10^−3^	24/156	27/66	1/4
**25:22038367**	**52/51**	36/48	16/3	0/0	**1.1 x 10** ^**−3**^	**175**	**8.2 x 10** ^**−6**^	36/209	16/16	0/1

^a^CanFam3.1 genomic location of the SNP in the format chromosome: base pair. SNPs showing evidence of replication are highlighted in bold.

^b^0, 1 and 2 copies of risk allele as per Table 1.

### Validation testing of a five-SNP genetic risk score

**Fig 2:**
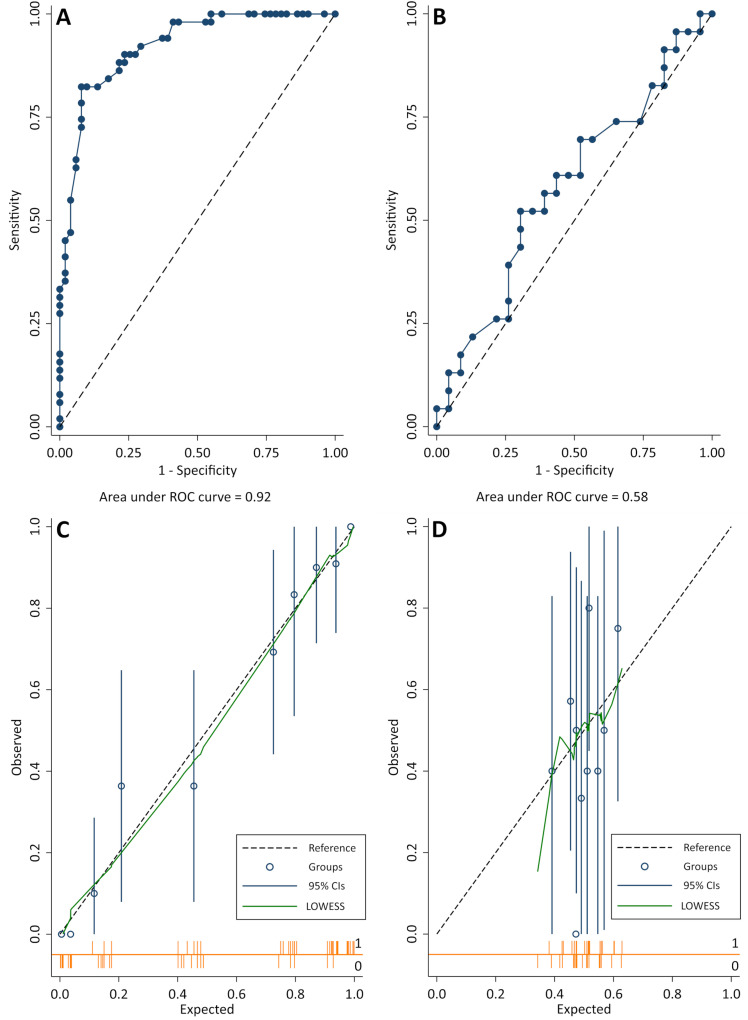
Receiver operating characteristic (ROC) curves and calibration plots for a five-SNP genetic risk score for idiopathic epilepsy in the Italian Spinone. Plots A and B are ROC curves for GWAS individuals, and the validation set, respectively. Points represent each potential genetic risk score cut-off for defining cases, from the highest (0,0) to the lowest (1,1). Sensitivity: fraction of cases correctly classified. Specificity: fraction of controls correctly classified (1 – (minus) specificity is the false-positive fraction). The area under the ROC curve (AUC) is given for each study set. An AUC of 0.5 (indicated by the dashed line) would represent a test unable to discriminate cases from controls. Calibration plots are shown for the GWAS (C), and replication set (D). Points represent ten equally sized groups of individuals divided by predicted risk. Observed: the proportion of cases in each group. Expected: the average (mean) of the predicted probabilities generated from the genetic risk score logistic regression model. The 95% confidence intervals are shown for each group. Orange lines are spike plots indicating the distribution of IE cases (1) and controls (0). The dashed reference line indicates perfect genetic risk score calibration where predicted risk matches the observed proportion of affected dogs within each group. Locally weighted scatterplot smoothing (LOWESS) is displayed in green.

To conduct further validation of SNPs showing increased statistical support for association with IE from the above analysis, and to assess the utility of a genetic risk score to predict a dog’s risk of developing IE, the five SNPs that showed an increased statistical association after the addition of the large control set were investigated as a weighted five-SNP genetic risk score in an independent validation set of 23 IE cases and 23 controls. Receiver operating characteristic (ROC) curve and calibration plot analysis demonstrated that the genetic risk score had poor predictiveness in the validation set (area under ROC curve (AUC): 0.58; 95% confidence intervals (CI): 0.41–0.75), compared with the GWAS discovery sets (AUC: 0.92; 95% CI: 0.87–0.97) (**Fig 2**). The validation set also failed to support the statistical associations of the five SNPs observed in the GWAS sets, both individually and as a five-SNP genetic risk score (**S4 Table**). A meta-analysis combining data from Set 1, 2 and the validation set was subsequently conducted and did not reveal any further associated loci (**S4 Fig**).

## Discussion

In the present study we used GWAS meta-analysis of 52 cases and 51 controls and subsequent replication analysis that aimed to identify regions of the genome showing reproducible association with IE in the Italian Spinone breed of dog. However, a genetic risk score comprised of five SNPs showing evidence of initial replication was not predictive for IE status in an independent validation set of 23 cases and 23 controls. Our findings demonstrate that IE in the Italian Spinone breed is not monogenic autosomal recessive or dominant, and that the mode of inheritance may be multigenic and/or complex. There could also be clinical heterogeneity in IE in the Spinone population, with underlying genetic heterogeneity, confounding GWAS studies and demanding much larger sample sizes to investigate the disease.

One potential explanation for the observed lack of replication is the differences in year of birth between the four sets of individuals studied meaning that the dogs could be multiple generations apart, potentially allowing genetic drift and selection by dog breeders to affect allele frequencies and changes in linkage disequilibrium. The Italian Spinone breed is not numerically large in the UK, with 350–526 (mean 453.9) Kennel Club registrations a year between 2012 and 2021 [22], and only a relatively small subset of these registered dogs is likely to be part of the breeding population. Changes in breeding strategies amongst dog breeders, for example avoiding dogs related to epilepsy cases, could therefore have the potential to quickly impact allele frequencies within the UK population. The requirement for controls to be a minimum of seven years old meant that there was only moderate overlap in year of birth between cases and controls for some study sets; Set 2 being the most extreme example of this. This sampling bias meant that cases and controls could be multiple generations apart, again suggesting that allele frequencies could have changed, with the potential of generating false positive results. However, MDS analysis did not show any clear stratification between the three array-genotyped sample sets, or cases and controls within each set, which would have provided evidence of sampling bias. The selection of ‘super controls’ with ages greater than the higher end of the range of the reported age of onset, as opposed to age-matched controls which could include dogs that would go on to have IE later in life, was a methodology chosen to increase study power and compensate in part for the small number of cases available [23]. The above factors were, however, likely still exacerbated by the small sample numbers used; the sample size was limited by the small numeric size of the breed which, along with the reportedly severe clinical course and high mortality rate [6], limits the number of IE cases present in the population at any given time. Small sample size would have reduced the study’s power to find associations with IE [23]. The validation set may have lacked sufficient power to replicate any true statistical associations with IE that were identified in the discovery GWAS due to the ‘winners curse’ phenomenon [23,24].

The findings demonstrate the importance of replicating associations identified through GWAS in an independent validation set [25]. Two of the regions identified in the GWAS (on chromosomes 2 and 8) passed the Bonferroni-adjusted threshold for statistical significance, accounting for multiple testing, and the region on chromosome 2 implicated a gene with compelling candidacy for epilepsy (*MAST4*) [26–29]. It is possible that this locus represents a higher effect, familial, variant that is present in a small number of dogs which has since been bred out of the population through the avoidance of breeding dogs closely related to cases, as discussed above. The risk allele frequencies suggest that the association observed in the GWAS meta-analysis was largely driven by Set 2 (**Table 1**); however, the validation set showed not only a reduction in risk allele frequency in cases but also an increase in risk allele frequency in controls. Whole genome sequencing (WGS) analysis of the different sets utilised in this study, and related individuals, could be used in future work to further examine this hypothesis.

It is important when intending to develop DNA-based breeding tools that they are based on robust evidence. This study is not the first to identify loci demonstrating statistically significant associations with canine IE in a discovery set that have not replicated in independent study sets. A locus on chromosome 14 demonstrated statistically significant association with IE in Belgian Shepherd dogs in a GWAS of 20 cases and 45 controls after correcting for multiple testing [30]. Subsequent investigation in larger independent case-control sets have failed to replicate the statistically significant association observed in the discovery set [31,32]. By comparison, a locus on chromosome 37 demonstrates replicable association with IE in the Belgian Shepherd dog and other breeds [25,30,33,34].

The GWAS approach cannot identify very common or fixed (i.e., present in all individuals) variants contributing to the breed’s overall increased risk of IE, or rare or *de novo* high-impact variants that may cause IE in an individual dog. The use of WGS datasets could be used to identify these variant types, complementing the existing GWAS data. An across-breed analysis could further help overcome this limitation by searching for loci, genes, and pathways that are important for IE risk in multiple breeds.

In conclusion, GWAS meta-analysis and replication analysis could not identify genomic loci with replicable association with IE in the Italian Spinone, independently or in a genetic risk score. However, this is the first reported study attempting to elucidate the underlying genetics of IE in this breed and will support future work aiming to gain an understanding of the aetiology of IE in the Italian Spinone and other breeds.

## Supporting information

S1 Fig Year of birth for cases and controls by study set.
Grey: Controls. Blue: Cases. Boxes indicate the lower to upper quartiles, and the white lines the median. Whiskers extend to the first datum beyond 1.5 times the interquartile range from the lower and upper quartiles. Circles indicate outliers. The plots share an x-axis.
(TIF)

S2 Fig MDS plot of the three study sets.
(TIF)

S3 Fig Forest plot showing the consistency of associations between study sets for the 12 most significantly associated SNPs from the meta-analysis. The forest plot was generated using Stata’s ‘metan’ command. SNP IDs are the CanFam3.1 genomic position in the format chromosome: base pair. Black diamonds are the odds ratio point estimates for each study. Grey box size indicates study weighting. Whiskers indicate lower and upper 95% confidence intervals of the odds ratio. ‘Subtotal’ green diamonds represent the odds ratio point estimate (centre) and the lower and upper 95% confidence intervals of the odds ratio (left and right points respectively) for the meta-analysis.
(TIF)

S4 Fig GWAS meta-analysis of 70 Italian Spinone idiopathic epilepsy cases and 69 controls (320,780 SNPs).
Plot of negative log (base 10) transformed P-values. X-axis is SNP location by chromosome (left to right, autosomes 1 to 38). Solid circles indicate array-genotyped SNPs, hollow triangles denote SNPs imputed for Set 1 or the replication set. Green (upper) line shows the Bonferroni-corrected threshold for statistical significance (P < 1.6 x 10^−7^). Orange (lower) line indicates the threshold for suggestive association (P < 1 x 10^−5^).
(TIF)

S1 Table Sample details including collection years, years of birth, country of origin, and method of case diagnosis.
(DOCX)

S2 Table Mean kinship coefficients among cases, controls, and random samples of the Kennel Club registered population.
(DOCX)

S3 Table Allelic discrimination assay primer and reporter probe sequences used to genotype the 10 SNPs identified from the GWAS meta-analysis of IE.
(DOCX)

S4 Table Results from analysis of the five-SNP genetic risk score SNPs individually and combined as a genetic risk score, and risk allele frequencies in cases and controls.
(DOCX)

S1 File Additional methodology and results: kinship calculations and selection of controls for Set 2.
(DOCX)
