# Supplementary figures and images for: Genome-wide association study of idiopathic epilepsy in the Italian Spinone dog breed
Source: PLoS One. 2025 Mar 5;20(3):e0315546. doi: 10.1371/journal.pone.0315546 (PMC11882058; doi:10.1371/journal.pone.0315546)

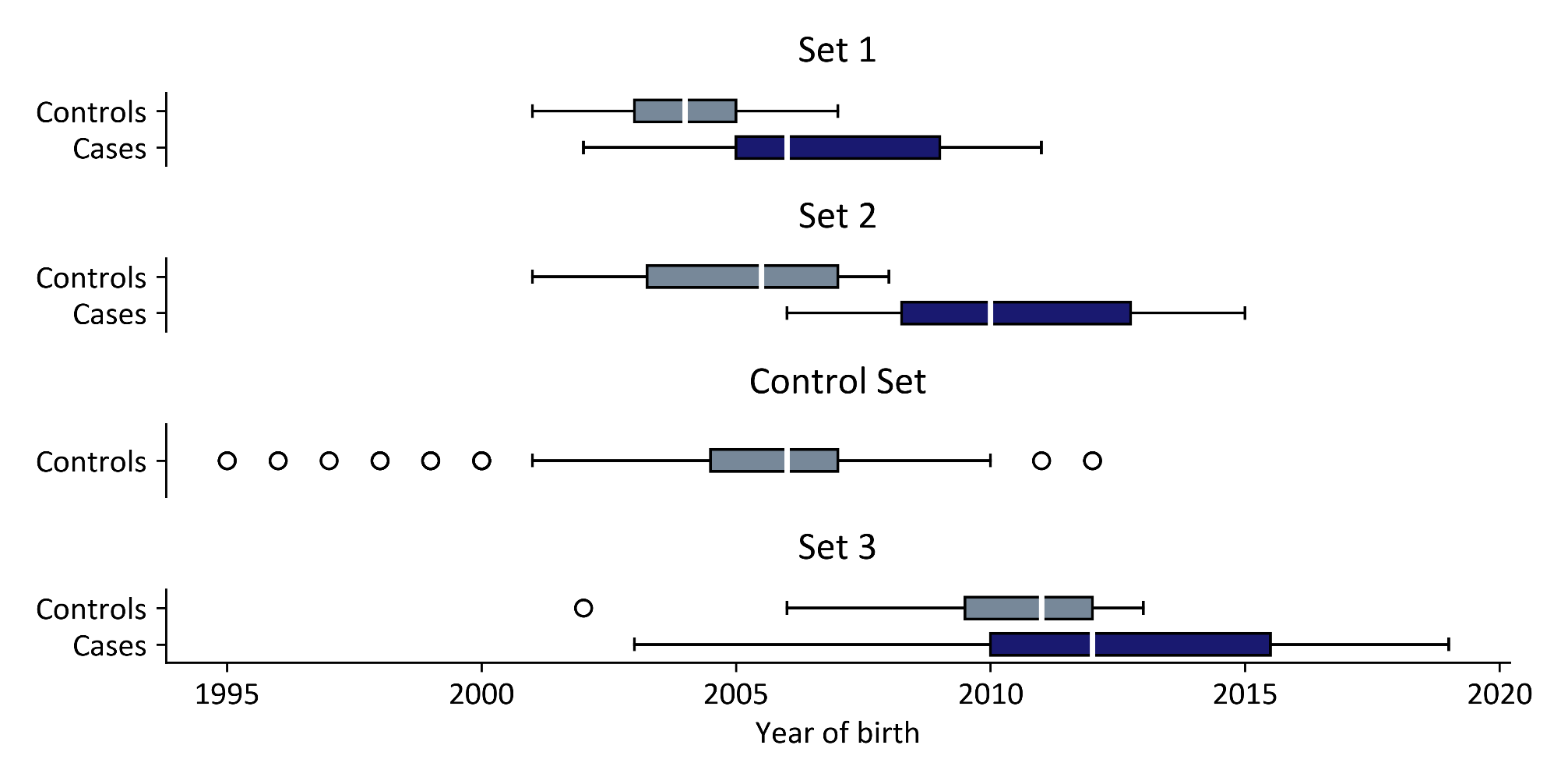

Supplement: S1 Fig — Grey: Controls. Blue: Cases. Boxes indicate the lower to upper quartiles, and the white lines the median. Whiskers extend to the first datum beyond 1.5 times the interquartile range from the lower and upper quartiles. Circles indicate outliers. The plots share an x-axis. (TIF) [file pone.0315546.s001.tif]

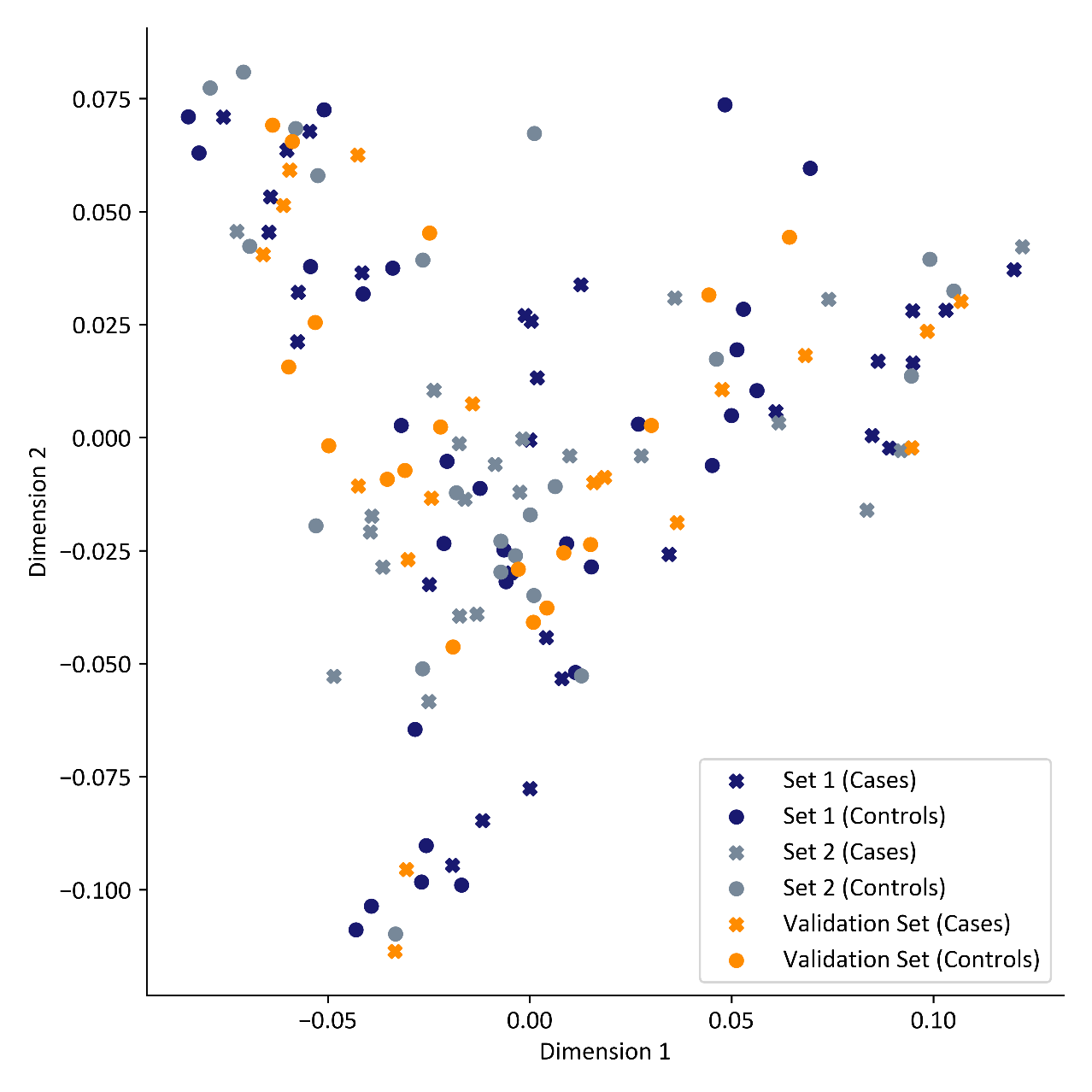

Supplement: S2 Fig — (TIF) [file pone.0315546.s002.tif]

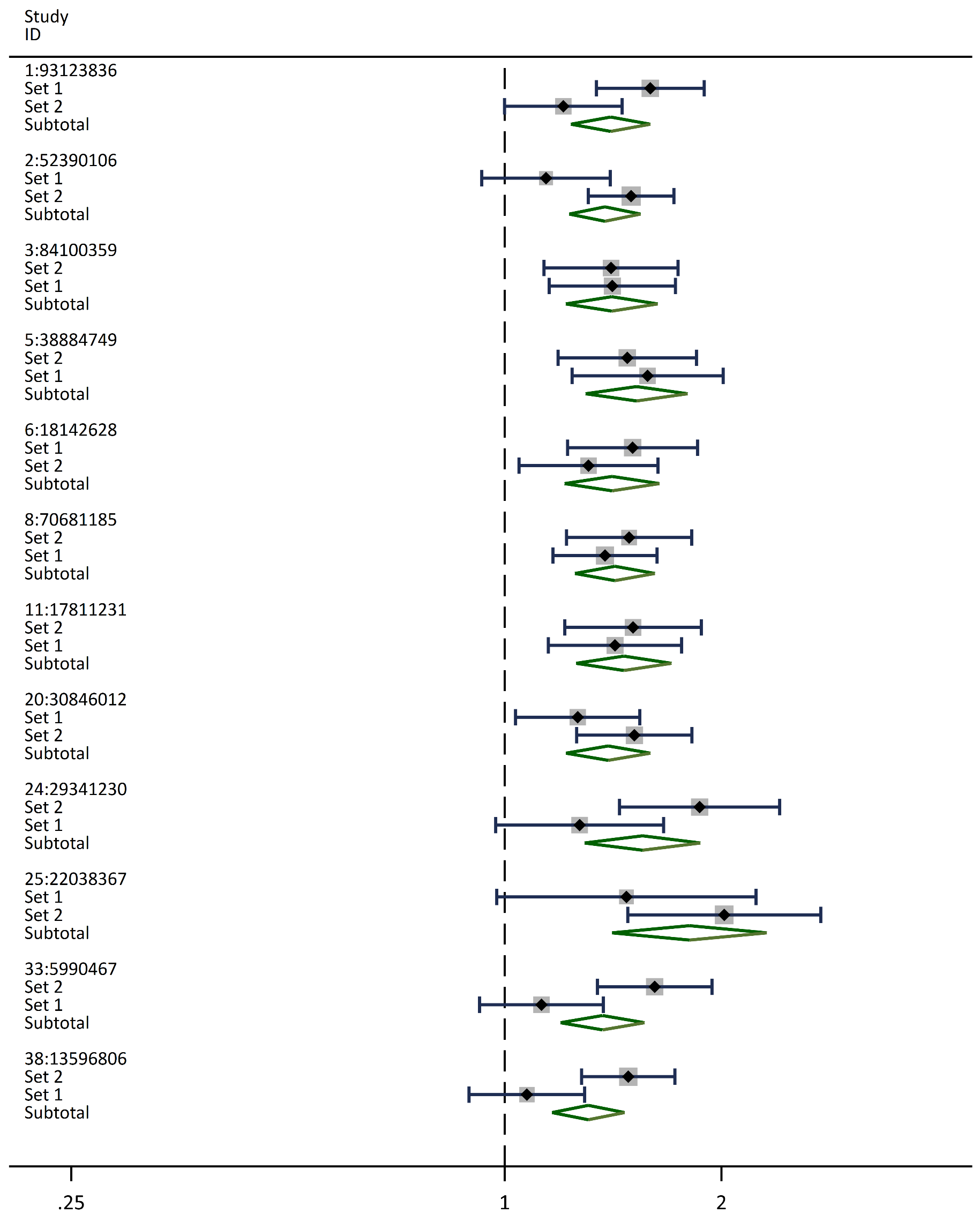

Supplement: S3 Fig — The forest plot was generated using Stata’s ‘metan’ command. SNP IDs are the CanFam3.1 genomic position in the format chromosome: base pair. Black diamonds are the odds ratio point estimates for each study. Grey box size indicates study weighting. Whiskers indicate lower and upper 95% confidence intervals of the odds ratio. ‘Subtotal’ green diamonds represent the odds ratio point estimate (centre) and the lower and upper 95% confidence intervals of the odds ratio (left and right points respectively) for the meta-analysis. (TIF) [file pone.0315546.s003.tif]

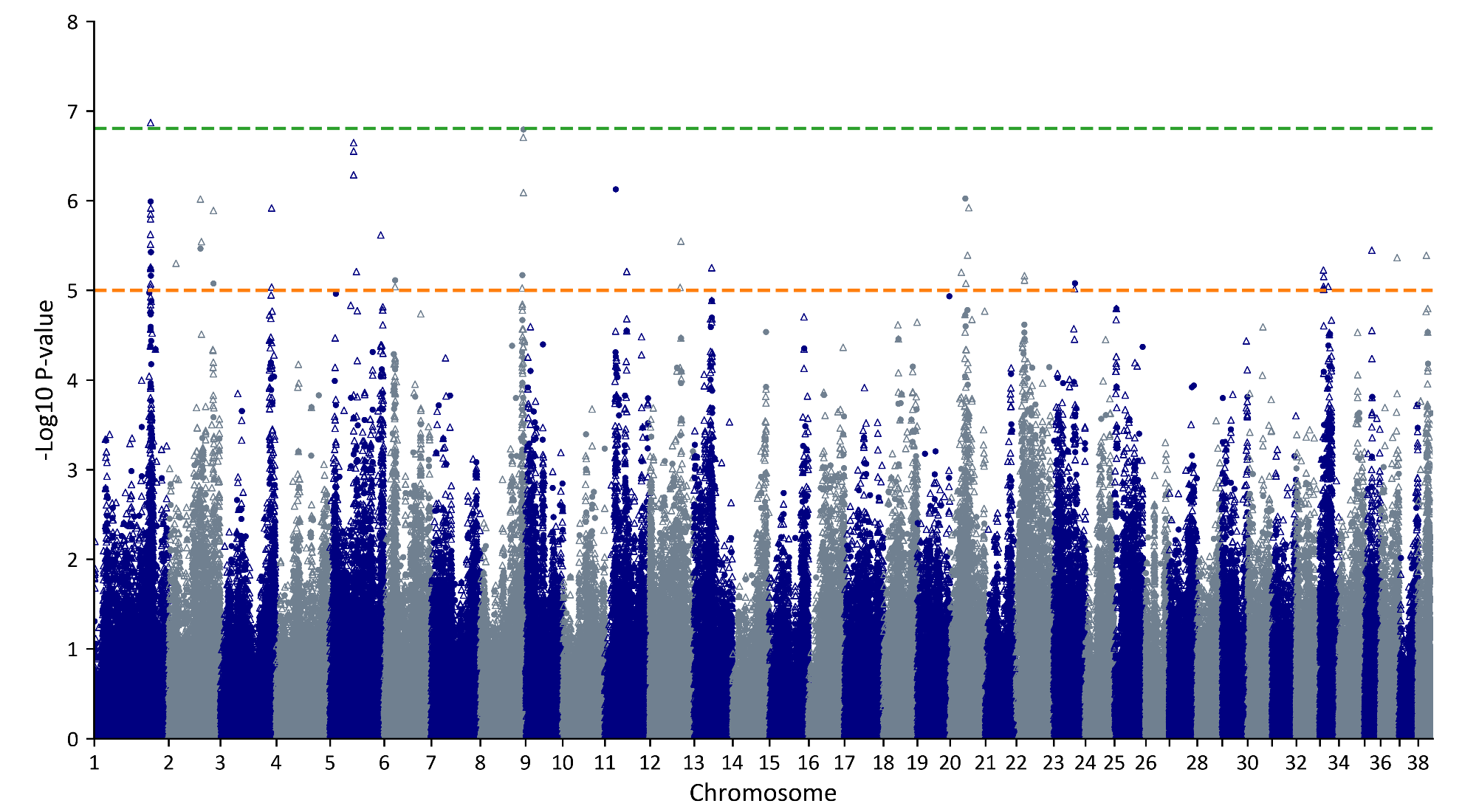

Supplement: S4 Fig — Plot of negative log (base 10) transformed P-values. X-axis is SNP location by chromosome (left to right, autosomes 1 to 38). Solid circles indicate array-genotyped SNPs, hollow triangles denote SNPs imputed for Set 1 or the replication set. Green (upper) line shows the Bonferroni-corrected threshold for statistical significance (P < 1.6 x 10^−7^). Orange (lower) line indicates the threshold for suggestive association (P < 1 x 10^−5^). (TIF) [file pone.0315546.s004.tif]
