# Supplementary material for: Genome-wide association study of idiopathic epilepsy in the Italian Spinone dog breed
Source: PLoS One. 2025 Mar 5;20(3):e0315546. doi: 10.1371/journal.pone.0315546 (PMC11882058; doi:10.1371/journal.pone.0315546)
Supplement: S1 Table — (DOCX) [file pone.0315546.s005.docx]

**S1 Table. Sample details including collection years, years of birth, country of origin, and method of case diagnosis**

|  |  | **Discovery sets** | | **Replication sets** | |
| --- | --- | --- | --- | --- | --- |
|  |  | **Set 1** | **Set 2** | **Control set** | **Validation set** |
| **Number of cases and controls** | | 29 cases, 29 controls | 23 cases, 22 controls | 175 controls | 23 cases, 23 controls |
| **Samples collected (year)** | | 2007-2014 | 2014-2017 | 2007-2019 | 2007-2021 |
| **Years of birth** | **Cases** | 2002-2011 | 2006-2015 (1 unknown) | - | 2003-2019 (4 unknown) |
|  | **Controls** | 2001-2007 | 2001-2008 | 1995-2012 | 2002-2013 |
| **Country of origin (n)** | | UK (49), USA (7), Australia (2) | UK (32), USA (12), Germany (1) | UK (118), USA (54), Italy (4), Netherlands (2), Belgium (1), Denmark (1), Romania (1) | UK (38), USA (4), Canada (2), Finland (2) |
| **Case diagnosis methodology** | | Breed-wide survey.  Animal Health Trust neurology unit. | Owner reported questionnaire and veterinary records, where available.  Animal Health Trust neurology unit, UK. | - | Owner reported questionnaire and veterinary records, where available.  Animal Health Trust neurology unit, UK.  Linnaeus referral veterinary hospitals, UK. Two samples from University of Helsinki, Finland |
| **Genotyping array/method** | | Illumina CanineHD BeadChip | Axiom Canine HD array | Allelic discrimination assays | Illumina CanineHD BeadChip |
