# Supplementary material for: Genome-wide association study of idiopathic epilepsy in the Italian Spinone dog breed
Source: PLoS One. 2025 Mar 5;20(3):e0315546. doi: 10.1371/journal.pone.0315546 (PMC11882058; doi:10.1371/journal.pone.0315546)
Supplement: S2 Table — (DOCX) [file pone.0315546.s006.docx]

**S2 Table. Mean kinship coefficients among cases, controls, and random samples of the Kennel Club registered population**

| **Mean kinship** | **Actual cases** | **Actual controls** | **Random sample (‘cases’)** | **Random sample (‘controls’)** |  | **95% lower CI** | **95% upper CI** |
| --- | --- | --- | --- | --- | --- | --- | --- |
| Actual cases | 0.0490 | - | - | - |  |  |  |
| Actual controls | 0.0464 | 0.0579 | - | - |  |  |  |
| Random sample (‘cases’) | 0.0404 | - | 0.0400 | - | Random samples 'cases' | 0.0284 | 0.0516 |
| Random sample (‘controls’) | - | 0.0569 | 0.0429 | 0.0548 | Random samples 'controls' | 0.0419 | 0.0678 |
|  |  |  |  |  | Between random samples | 0.0333 | 0.0525 |

Mean kinship coefficients among and between dogs within actual case and control cohorts, and the mean of 1,000 average kinship coefficients among and between samples of n=21 (‘cases’) or n=25 (‘control’) randomly selected dogs, and between these samples and actual case and control cohorts. CI = confidence interval.
