# Supplementary material for: Genome-wide association study of idiopathic epilepsy in the Italian Spinone dog breed
Source: PLoS One. 2025 Mar 5;20(3):e0315546. doi: 10.1371/journal.pone.0315546 (PMC11882058; doi:10.1371/journal.pone.0315546)
Supplement: S3 Table — (DOCX) [file pone.0315546.s007.docx]

**S3 Table.** **Allelic discrimination assay primer and reporter probe sequences used to genotype the 10 SNPs identified from the GWAS meta-analysis of IE**

| **Genomic position*** | **Forward Primer** | **Reverse Primer** | **Reporter 1 (VIC)** | **Reporter 2 (FAM)** |
| --- | --- | --- | --- | --- |
| 1:93123836 | AGCACAAAGGAACAAGCTAAACCT | TGCAATGCTAGTGACACTGTGT | CAGAGGCTTTAGACGTGCT | CAGAGGCTTTAAACGTGCT |
| 2:52390106 | CTCACCTTACCCATTTGCTTTTGTG | GGGAGCCATAGTTAAATGAAATGTGATCT | CAAACTTATCCTGTTCTTTGAGAT | AACTTATCCTGTTCCTTGAGAT |
| 3:84100359 | TGTAGTACTGTAGCAGATGTATCATGAGTTATAA | CCAAGGCTGGGAGATAAATACCAAT | CTGGGAACAGTCAGTTTA | TGGGAACAGTCACTTTA |
| 5:38884749 | GCTGCGCGTTCACTTTGG | TCCAGGCTGATTCATCATTGTTACC | CCAAATTTACAGATAACAATAT | AAATTTACAGATGACAATAT |
| 6:18142628 | GCTCAGCAATGCCAGAGACATAAA | ACTAGCAGCAGATAAGCTCTGTCT | TTAGATTACACGACAGATTT | ATTACACGGCAGATTT |
| 8:70681185 | TCCATAGAGTTAGGACCCCTTGTG | CAGCTTCTGTCCATATGTGAAGTCAA | TGCTTTTCTACAAGCGTTTG | TGCTTTTCTACAATCGTTTG |
| 11:17811231 | TGATACATAAAGTAAGCAAGGGAGATCCA | ACCACACCCTTTTTCATTGCAAAAT | CAAAGTGTGTCTTTTAACCAG | AAGTGTGTCTTCTAACCAG |
| 20:30846012 | CGATTGTGTCCAAAGAAGGAATGTC | GACCAAGATCTGTCCTTGAAAATGTC | CAACTTATGGTTTGCTTTAA | ACTTATGGTTCGCTTTAA |
| 24:29341230 | AATTGGGTGTCATTTATTTATTTATTTTTGCTAAA | GTCTGGCTCCCTCTAGTATGGA | CATCAACGGTCTGACCC | TCAACGGCCTGACCC |
| 25:22038367 | CAAGGCACACTAAGCAGACCAT | GGATCCCTCATAGTCCATAATAGAAAAGT | TAGCCTCACTTTATGGTTC | TAGCCTCACTTTTTGGTTC |

*CanFam3.1 in the format: chromosome: base pair
