# Supplementary material for: Genome-wide association study of idiopathic epilepsy in the Italian Spinone dog breed
Source: PLoS One. 2025 Mar 5;20(3):e0315546. doi: 10.1371/journal.pone.0315546 (PMC11882058; doi:10.1371/journal.pone.0315546)
Supplement: S4 Table — (DOCX) [file pone.0315546.s008.docx]

**S4 Table. Results from analysis of the five-SNP genetic risk score SNPs individually and combined as a genetic risk score, and risk allele frequencies in cases and controls.**

|  | **Set 1** | | **Set 2** | | **GWAS** | | **Validation set** | | | |
| --- | --- | --- | --- | --- | --- | --- | --- | --- | --- | --- |
| **Genomic Pos. *^a^*** | **ca/co *^b^*** | **Risk Allele Freq. (ca/co *^b^*)** | **ca/co *^b^*** | **Risk Allele Freq. (ca/co *^b^*)** | **OR (95% CI)** | **P-value** | **OR (95% CI)** | **P-value** | **ca/co *^b^*** | **Risk Allele Freq. (ca/co *^b^*)** |
| 1:93123836 | 29/29 | 0.84/0.47 | 22/22 | 0.68/0.43 | 4.61  (2.28 - 9.32) | 1.5 x 10^-6^ | 1.45  (0.62 - 3.42) | 0.39 | 23/23 | 0.59/0.50 |
| 2:52390106 | 29/29 | 0.50/0.34 | 22/22 | 0.70/0.16 | 3.38  (1.86 - 6.16) | 1.3 x 10^-5^ | 1.00  (0.45 - 2.24) | 1.00 | 23/23 | 0.46/0.46 |
| 11:17811231 | 29/29 | 0.84/0.62 | 22/22 | 0.91/0.61 | 5.64  (2.47 - 12.86) | 5.3 x 10^-6^ | 1.59  (0.61 - 4.17) | 0.34 | 23/23 | 0.78/0.70 |
| 20:30846012 | 29/29 | 0.69/0.50 | 22/22 | 0.75/0.36 | 4.57  (2.13 - 9.80) | 9.9 x 10^-6^ | 1.27  (0.58 - 2.81) | 0.55 | 23/23 | 0.52/0.46 |
| 25:22038367 | 29/29 | 0.10/0.05 | 22/22 | 0.20/0.00 | 6.72  (1.80 - 25.05) | 1.2 x 10^-3^ | 0.45  (0.07 - 2.76) | 0.38 | 23/23 | 0.04/0.09 |
| **Five-SNP genetic risk score** | 29/29 | - | 22/22 | - | 5.69  (2.97 - 10.90) | 5.2 x 10^-17^ | 1.23  (0.75 - 2.00) | 0.40 | 23/23 | - |

*^a^* CanFam 3.1 genomic location of the SNP in the format chromosome: bp position. Genomic positions shown in bold indicate the most significantly associated SNPs in the GWAS meta-analysis. *^b^* case / control. Only individuals with genotype data for all five SNPs were included in the analysis.
