# Supplementary material for: Genome-wide association study of idiopathic epilepsy in the Italian Spinone dog breed
Source: PLoS One. 2025 Mar 5;20(3):e0315546. doi: 10.1371/journal.pone.0315546 (PMC11882058; doi:10.1371/journal.pone.0315546)
Supplement: S1 File — (DOCX) [file pone.0315546.s009.docx]

Due to the selection criteria requiring control dogs be over the age of seven years, the cases and controls in Set 2 had only partially overlapping years of birth. Therefore, two separate random sample sets were used in the analysis of the Set 2 case-control set, one comprising n=21 dogs born between 2008 and 2016 mirroring the ‘cases’, and the other comprising 25 dogs born between 2001 and 2009 as per the controls.

Relationships between each pair of dogs in a particular cohort were determined using kinship coefficients (the kinship between two individuals is equal to the inbreeding coefficient of their offspring). Ancestors of the dogs in each cohort were isolated from the Kennel Club pedigree, and ‘dummy’ progeny were created for each pair of individuals in the cohort. Inbreeding coefficients for the ‘dummy’ progeny (i.e., kinship coefficients of each pair) were calculated utilising the algorithm of Meuwissen and Luo (1992) using a script written in MATLAB.

The mean kinships among and between the ‘cases’ and ‘controls’ cohorts used in this study were representative of random samples of dogs from similar birth years (**S2 Table**).

Standard deviations of kinship coefficients amongst the cases (0.0513); controls (0.0578); and between (0.0464) were within but at the higher end of the 95% confidence intervals from the 1,000 random samples: 0.0233–0.0515 for samples representing ‘cases’; 0.0339–0.0593 in samples representing ‘controls, and 0.0266–0.0486 between. A possible reason for this comparatively higher variance in pair-wise kinships among actual case and control cohorts is the close relationships between a small number of dogs therein (full-siblings and parent/progeny).

Reference

Meuwissen THE, Luo Z. Computing inbreeding coefficients in large populations. Genetics Selection Evolution. 1992;24(4):305. doi: 10.1186/1297-9686-24-4-305.
